# Selective nitration of Hsp90 acts as a metabolic switch promoting tumor cell proliferation

**DOI:** 10.1016/j.redox.2024.103249

**Published:** 2024-06-19

**Authors:** Isabelle E. Logan, Kyle T. Nguyen, Tilottama Chatterjee, Bhagyashree Manivannan, Ngozi P. Paul, Sharon R. Kim, Evelyn M. Sixta, Lydia P. Bastian, Carrie Marean-Reardon, Matthias A. Karajannis, Cristina Fernández-Valle, Alvaro G. Estevez, Maria Clara Franco

**Affiliations:** a Department of Biochemistry and Biophysics, College of Science, Oregon State University, Corvallis, OR, 97331, USA; b Center for Translational Science, Florida International University, Florida, 34987, USA; c Department of Pediatrics, Memorial Sloan Kettering Cancer Center, New York, NY, 10065, USA; d Burnett School of Biomedical Sciences, College of Medicine, University of Central Florida, Orlando, FL, 32827, USA; e Department of Cellular and Molecular Medicine, Herbert Wertheim College of Medicine, Florida International University, Florida, 33199, USA

**Keywords:** Neurofibromatosis, Tyrosine nitration, Tumor, Hsp90, P2X7 receptor, Cell metabolism

## Abstract

Tumors develop in an oxidative environment characterized by peroxynitrite production and downstream protein tyrosine (Y) nitration. We showed that tyrosine nitration supports schwannoma cell proliferation and regulates cell metabolism in the inheritable tumor disorder *NF2*-related Schwannomatosis (NF2-SWN). Here, we identified the chaperone Heat shock protein 90 (Hsp90) as the first nitrated protein that acts as a metabolic switch to promote schwannoma cell proliferation. Doubling the endogenous levels of nitrated Hsp90 in schwannoma cells or supplementing nitrated Hsp90 into normal Schwann cells increased their proliferation. Metabolically, nitration on either Y33 or Y56 conferred Hsp90 distinct functions; nitration at Y33 (Hsp90_NY33_) down-regulated mitochondrial oxidative phosphorylation, while nitration at Y56 (Hsp90_NY56_) increased glycolysis by activating the purinergic receptor P2X7 in both schwannoma and normal Schwann cells. Hsp90_NY33_ and Hsp90_NY56_ showed differential subcellular and spatial distribution corresponding with their metabolic and proliferative functions in schwannoma three-dimensional cell culture models. Collectively, these results underscore the role of tyrosine nitration as a post-translational modification regulating critical cellular processes. Nitrated proteins, particularly nitrated Hsp90, emerge as a novel category of tumor-directed therapeutic targets.

## Highlights

• Selective tyrosine nitration of Hsp90 induces cell proliferation through activation of P2X7R.
• Nitrated Hsp90 acts as a metabolic switch in tumor cells.
• Site-specific nitration of Hsp90 at Y33 decreases mitochondrial metabolism.
• Site-specific nitration of Hsp90 at Y56 increases glycolysis.
• Nitrated Hsp90 is the first in a novel category of tumor-directed therapeutic targets.

## Graphical abstract

**Figure:**
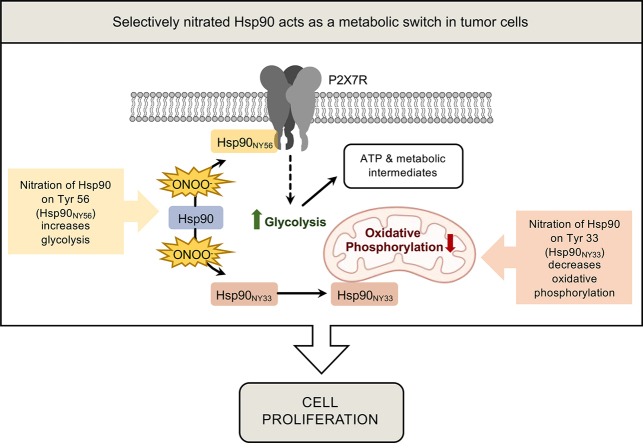

## 1 Introduction

Inactivating mutations in the *NF2* gene, which codes for the tumor suppressor merlin, causes the autosomal-dominant tumor predisposition disorder of the nervous system *NF2*-related Schwannomatosis (NF2-SWN, formerly known as neurofibromatosis type 2) [[1], [2], [3]]. NF2-SWN affects approximately one in twenty-five thousand individuals worldwide, who develop multiple benign tumors, including schwannomas, meningiomas, and ependymomas throughout their life [[4], [5], [6], [7]].

In pathological conditions associated with oxidative stress, such as those found in solid tumors [8,9], the reaction of nitric oxide (^.^NO) with superoxide (O_2_^.-^) forms the potent oxidant peroxynitrite (ONOO^−^). Peroxynitrite production then leads to irreversible protein tyrosine nitration [10,11]. Historically, peroxynitrite production and tyrosine nitration were used as oxidative stress markers and correlated with cell death [[11], [12], [13], [14], [15]]. We and others showed that nitrated proteins are not just byproducts of oxidative stress but play definitive pathological roles in disease processes. Depending on the location of the tyrosine residue, nitration changes protein function, leading to functional activation or inactivation, or even inducing a new function the normal protein cannot perform. For example, nitration of Y34 in the active site of the mitochondrial enzyme manganese superoxide dismutase (MnSOD) inhibits its antioxidant activity in chronic kidney rejection [16,17]. On the other hand, in pulmonary hypertension, activation of the small GTPase transforming protein RhoA by nitration on Y34 enhances mitochondrial fission and increases glycolysis in pulmonary arterial endothelial cells, leading to a dysfunctional pulmonary vasculature [18]. We discovered that selective nitration of the molecular chaperone Heat shock protein 90 (Hsp90) has different effects depending on the nitrated residue and the cells type. While nitration of Hsp90 on Y56 turns the pro-survival protein into a mediator of motor neuron and PC12 cells death through a gain-of-function, nitration on Y33 induces motor neuron death but leads to decreased mitochondrial metabolism in PC12 cells [[19], [20], [21]].

Nitrated proteins are also detected in various tumor types, including pancreatic ductal adenocarcinoma, breast and colon cancer, glioblastoma, and schwannomas [[22], [23], [24], [25], [26], [27]]. In metastatic melanoma and urinary bladder carcinoma, the nitrotyrosine levels correlate with poor prognosis [28,29], suggesting that nitrated proteins may play a proliferative role in tumor biology. We previously described that merlin deficiency leads to increased peroxynitrite production and tyrosine nitration in schwannoma cells, and that nitrated proteins selectively support cell survival and/or proliferation in both mouse and human schwannoma cell culture models [27]. In these tumor cells, peroxynitrite production and subsequent tyrosine nitration causes a metabolic shift from mitochondrial oxidative phosphorylation towards glycolysis and glutaminolysis, a hallmark of tumor cell metabolism [27]. Collectively, these observations suggest that one or more nitrated proteins regulate tumor cell metabolism to increase cell proliferation.

Hsp90 is an abundant and essential molecular chaperone required for the proper folding and activity of more than 200 client proteins, including pro-survival and pro-apoptotic proteins, transcription factors and proteins involved in cell signaling [[30], [31], [32], [33], [34], [35], [36], [37]]. Further, Hsp90 regulates gene expression [38,39] and aids in the translocation of proteins to the mitochondria [[40], [41], [42], [43]]. The primary sequence of human Hsp90 contains 24 tyrosine residues, 5 of which we described are prone to nitration, located at positions 33, 56, 276, 484 and 596 [27]. In motor neurons and PC12 cells, we determined that nitration of Hsp90 at Y33 and/or Y56 is sufficient and necessary to induce a pathological gain-of-function [[19], [20], [21]]. While Hsp90 nitrated at Y56 induces motor neuron and PC12 cell death, Hsp90 nitrated at Y33 induces motor neuron death but in PC12 cells, the nitrated protein localizes in the mitochondrial outer membrane, forming a protein complex that down-regulates mitochondrial activity [21]. This intracellular location and activity suggest that in tumor cells, nitrated Hsp90 could play a critical metabolic role. Here, we identified nitrated Hsp90 as a metabolic switch and the first proliferative nitrated target in schwannoma cells. Intracellular delivery of nitrated Hsp90 increased the proliferation of normal Schwann cells and recapitulated the metabolic phenotype observed in schwannoma cells. Further, we identified the critical tyrosine residues responsible for the metabolic and proliferative activity of nitrated Hsp90. Doubling the endogenous levels of Hsp90 nitrated site-specifically at either Y33 or Y56 in schwannoma cells increased their proliferation and supplementing these different forms of nitrated Hsp90 in normal Schwann cells had a similar effect. Metabolically, nitrated Hsp90 acted as a metabolic switch, decreasing mitochondrial oxidative phosphorylation activity when nitrated at Y33, and increasing glycolysis when nitrated at Y56. The observed increase in glycolysis and cell proliferation in schwannoma cells was prevented by inhibiting the activity of the purinergic receptor P2X7 (P2X7R), suggesting that the receptor mediates nitrated Hsp90 metabolic and proliferative activity. Interestingly, not only did different forms of nitrated Hsp90 play distinct cellular and metabolic roles, their spatial distribution within the three-dimensional structure of schwannoma cell clusters differed, with increased levels of Hsp90 nitrated at Y33 in the outer cellular layer of the cell clusters, and Hsp90 nitrated at Y56 homogenously distributed throughout the three-dimensional structure. We demonstrate here that a nitrated protein can play critical roles in major cellular processes such as energy metabolism and regulation of tumor cell proliferation. Moreover, distinct forms of nitrated Hsp90 perform complementary functions to tightly regulate cellular metabolism in tumor cells. These observations highlight the relevance of nitrated proteins as signaling molecules controlling critical cellular processes in pathological conditions. Because the levels of nitrated proteins in healthy tissues are low or undetectable, drugs that selectively and specifically target nitrated proteins may exhibit minimal side effects. Nitrated Hsp90 is the first identified member of this novel category of therapeutic tumor-directed targets.

## 2 Results

### 2.1 Site-specifically nitrated Hsp90 increases schwannoma cell proliferation

**Fig. 1:**
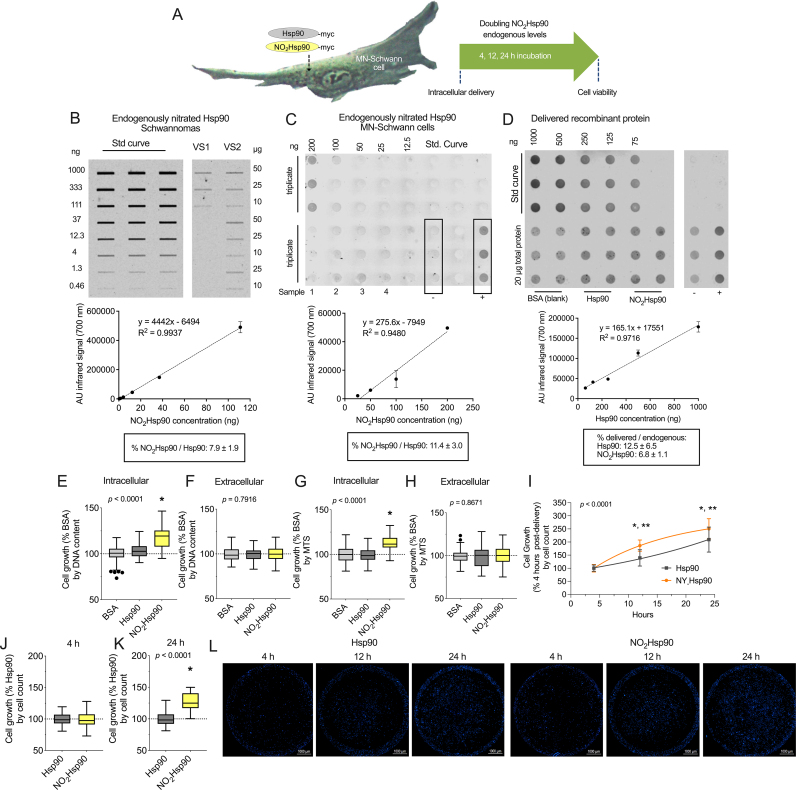
**Intracellular delivery of nitrated Hsp90 increases schwannoma cell proliferation.** (A) Summary of the experimental design. (B–D) Endogenous nitrated Hsp90 levels in (B) vestibular schwannomas (VS), and (C) schwannoma cells were determined by quantitative slot or dot blot using recombinant NO_2_Hsp90 for the standard (Std) curve and antibodies against Hsp90 nitrated at Y56. (D) Levels of protein intracellularly delivered were determined by quantitative dot blot 24 h post-delivery using recombinant Hsp90 for the Std curve and an antibody against the myc-tag. (E–H) Growth of schwannoma cells assessed by DNA content (E–F) or MTS assay (G–H), 24 h following protein delivery in the presence (intracellular) or absence (extracellular) of Chariot delivery reagent. (I) Schwannoma cell proliferation was assessed by cell count after DAPI staining at 4, 12, and 24 h post-intracellular delivery of Hsp90 or NO_2_Hsp90, and expressed as percentage of its corresponding 4-h timepoint (immediately after cell attachment). (J) Growth of schwannoma cells assessed by cell count after DAPI staining 24 h post-intracellular protein delivery, expressed as percentage of unmodified Hsp90. The cell growth curves were adjusted to an exponential growth (Hsp90) or exponential plateau (NO_2_Hsp90) model (n = 3–5 with 8–16 replicates). (K) Representative fluorescence pictures (40X) of wells containing schwannoma cells stained with DAPI 4, 12, and 24 h post-intracellular delivery of Hsp90 or NO_2_Hsp90. Scale bar represents 1 mm. In the plots, data is shown using Tukey's representation with the median indicated in the box plot and outliers indicated with dots (n = 5 with 8–16 replicates). *p < 0.0001 *versus* Hsp90 and **p < 0.0001 *versus* the earlier timepoint by one-way or two-way ANOVA with Tukey's multiple comparisons post hoc test.

We previously showed that peroxynitrite and tyrosine nitration support schwannoma cell survival and proliferation but the nitrated protein(s) playing this supportive role were unknown. We found Hsp90 endogenously nitrated in schwannoma cells and in resected tumors from NF2-SWN patients [27], suggesting that in tumor cells, nitrated Hsp90 may play a proliferative function. To investigate the role of nitrated Hsp90 in schwannoma cell proliferation, we doubled the endogenous levels of nitrated Hsp90 by intracellular delivery of peroxynitrite-treated recombinant Hsp90 (fully nitrated protein, NO_2_Hsp90) in schwannoma cells (Fig. 1A). We first determined the levels of endogenously nitrated Hsp90 *versus* total Hsp90 in schwannoma cells and in tumors from NF2-SWN patients using selective and highly specific antibodies we developed against Hsp90 nitrated at either Y33 or Y56 [[19], [20], [21],^27^]. Hsp90 nitrated on Y56 (Hsp90_NY56_) represented 7.9 ± 1.9 % of total Hsp90 in NF2-SWN vestibular schwannomas (VS; Fig. 1B and Sup. Figs. 1A), and 11.4 ± 3.0 % in schwannoma cells in culture (Fig. 1C, Sup. Fig. 1B). Hsp90 nitrated on Y33 (Hsp90_NY33_) represented 12.3 ± 6.7 % of total Hsp90 in NF2-SWN VS (Sup. Fig. 2A), and approximately 1.0 % in schwannoma cells in culture (Sup. Fig. 2B). As the stoichiometry of NO_2_Hsp90 to native Hsp90 in NF2-SWN tumors and cell culture model was approximately 1:10, we optimized the intracellular delivery protocol to introduce similar levels of recombinant NO_2_Hsp90 into schwannoma cells (Fig. 1D, Sup. Fig. 1C). Doubling the levels of NO_2_Hsp90 induced a statistically significant increase in cell growth 24 h post-delivery, while incubation of the cells with extracellular NO_2_Hsp90, in the absence of the delivery agent, had no effect, either by measuring DNA content (Fig. 1E and F) or via MTS assays (Fig. 1G and H). To confirm the increase in cell growth was due to enhanced cell proliferation, the cells were stained with DAPI and counted at 4, 12, and 24 h post-delivery. Intracellular delivery of NO_2_Hsp90 significantly increased cell numbers at 12 and 24 h post-delivery versus delivery of Hsp90, expressed as percentage of the cell numbers present 4 h post-delivery, after cell attachment to the plate (Fig. 1I–K and Sup. Fig. 1D). Collectively, these results suggest that doubling the endogenous NO_2_Hsp90 levels increases schwannoma cell proliferation.

**Fig. 2:**
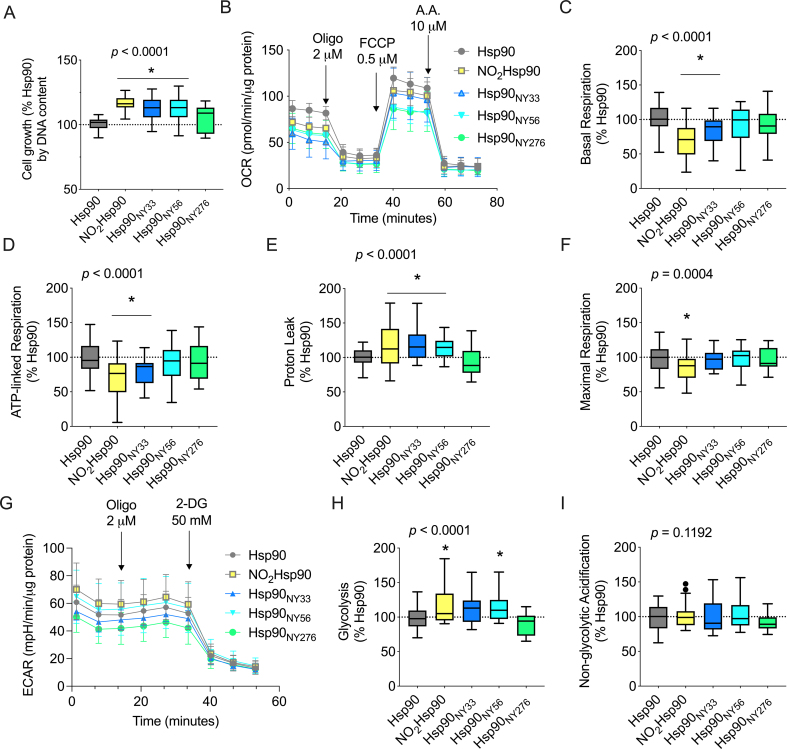
**Site-specifically nitrated Hsp90 decreases mitochondrial activity and increases glycolysis in schwannoma cells.** (A) Schwannoma cell growth was assessed following intracellular delivery of site-specifically nitrated Hsp90 24 h after delivery. (B) Representative experiment showing the oxygen consumption rate (OCR) of schwannoma cells 24 h post-intracellular protein delivery using the permeant agent Chariot, measured by extracellular flux analysis in basal conditions and following the sequential addition of oligomycin (2 μM, oligo), FCCP (0.5 μM), and antimycin A (AA, 10 μM). (C–F) Analysis of the different OCR parameters following the sequential addition of the inhibitors. (G) Representative experiment showing the extracellular acidification rate (ECAR) of schwannoma cells 24 h post-intracellular protein delivery, measured by extracellular flux analysis in basal conditions and following the sequential addition of oligomycin (2 μM, oligo) and 2-deoxyglucose (50 mM, 2-DG). (H–I) Analysis of the different ECAR parameters following the sequential addition of the inhibitors, expressed as the percentage of the intracellular delivery of Hsp90. Data is shown using Tukey's representation with the median indicated in the box plot and outliers indicated with dots (n = 5 with 8 replicates). *p < 0.005 *versus* Hsp90 by one-way ANOVA with Dunnett's multiple comparisons post hoc test.

### 2.2 Delivery of site-specifically nitrated Hsp90 into schwannoma cells reprograms their metabolism and increases cell proliferation

To determine whether nitration at Y33 or Y56 was relevant to the proliferative activity of nitrated Hsp90 in schwannoma cells, we used genetic code expansion (GCE) to incorporate the noncanonical amino acid nitrotyrosine site-specifically as the sole oxidative modification in the recombinant protein at positions 33 (Hsp90_NY33_), 56 (Hsp90_NY56_), or 276 (Hsp90_NY276_, used as a nitrated control). While intracellular delivery of Hsp90_NY276_ had no apparent proliferative effect (Fig. 2A), similar to the effect observed for NO_2_Hsp90, nitration of either Y33 or Y56 in Hsp90 was sufficient to induce a proliferative gain-of-function in schwannoma cells 24 h post-delivery (Fig. 2A).

We showed that Hsp90 nitrated at Y33 localizes to mitochondria and down-regulates mitochondrial metabolism in PC12 cells from a rat pheochromocytoma [21]. We also reported that nitrated Hsp90 co-localizes with mitochondria in schwannoma cells [27]. To determine a potential metabolic role for nitrated Hsp90 in schwannomas, we delivered recombinant NO_2_Hsp90 into schwannoma cells and measured the effect on mitochondrial metabolism. Using extracellular flux analysis, we found that doubling the intracellular levels of nitrated Hsp90 decreased all mitochondrial parameters, determined by assessing the oxygen consumption rate (OCR). Intracellular delivery of NO_2_Hsp90 significantly decreased basal and ATP-linked respiration (Fig. 2B–D), increased proton leak (Fig. 2E), and decreased maximal respiration (Fig. 2F) as compared to the delivery of Hsp90. To identify the tyrosine residues responsible for the regulation of mitochondrial activity, we delivered site-specifically nitrated Hsp90. Hsp90 nitrated at Y33 recapitulated the effects observed for NO_2_Hsp90, significantly decreasing both mitochondrial basal and ATP-linked respiration (Fig. 2C and D) and increasing proton leak (Fig. 2E). When nitrated at Y56, only proton leak increased compared to the delivery of Hsp90 (Fig. 2E). Intracellular delivery of Hsp90_NY276_ had no effect on mitochondrial activity in schwannoma cells (Fig. 2B–F). Collectively, these results suggest that nitration at Y33 is critical to Hsp90 mitochondrial gain-of-function.

We showed that peroxynitrite production in schwannoma cells leads to increased glycolytic activity [27]. To determine whether nitrated Hsp90 could act as a metabolic switch, decreasing mitochondrial ATP production in favor of glycolysis, we next assessed the glycolytic activity of schwannoma cells after delivery of the recombinant proteins. Only NO_2_Hsp90 and Hsp90_NY56_ increased glycolysis (Fig. 2G and H), while none of the delivered proteins influenced non-glycolytic acidification (Fig. 2I), suggesting that in contrast to the mitochondrial activity of nitrated Hsp90, nitration at Y56 is sufficient to induce a glycolytic regulatory function.

### 2.3 Nitration of Hsp90 triggers schwann cell proliferation

**Fig. 3:**
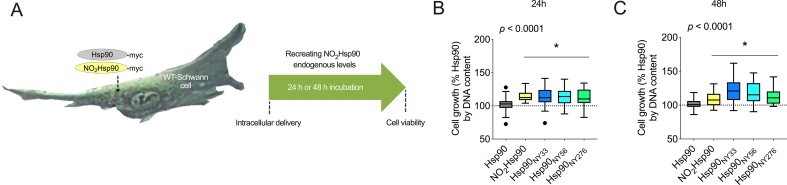
**Intracellular delivery of site-specifically nitrated Hsp90 increases normal Schwann cell proliferation.** (A) Summary of the experimental design. Growth of Schwann cells was assessed (B) 24 h or (C) 48 h following protein delivery. Data is shown using Tukey's representation with the median indicated in the box plot and outliers indicated with dots (n = 8 with 8 replicates). *p < 0.05 (NO_2_Hsp90) or *p* < 0.0001 (Hsp90_NY33_ and Hsp90_NY56_) *versus* Hsp90 by one-way ANOVA with Dunnett's multiple comparisons post hoc test.

To investigate whether intracellular delivery of either NO_2_Hsp90, Hsp90_NY33_, or Hsp90_NY56_ into normal Schwann cells, which do not contain detectable levels of endogenously nitrated Hsp90 [27], recapitulated the enhanced proliferative phenotype observed in schwannoma cells, we delivered these recombinant proteins into Schwann cells at levels comparable to those endogenously found in VS from NF2-SWN patients and schwannoma cells (Fig. 1), and determined cell growth 24 and 48 h post-delivery (Fig. 3A). Intracellular delivery of NO_2_Hsp90, Hsp90_NY33_, or Hsp90_NY56_ significantly increased Schwann cell proliferation compared to the delivery of Hsp90 both 24 h (Figs. 3B) and 48 h post-delivery (Fig. 3C). However, in contrast to the observation in schwannoma cells, intracellular delivery of Hsp90_NY276_ also increased proliferation (Fig. 3B and C), suggesting that nitration of Hsp90 at Y276 may also be relevant to schwannoma cell proliferation.

### 2.4 Delivery of fully oxidized or site-specifically nitrated Hsp90 into human schwann cells recapitulates the metabolic phenotype of schwannoma cells and increases proliferation

**Fig. 4:**
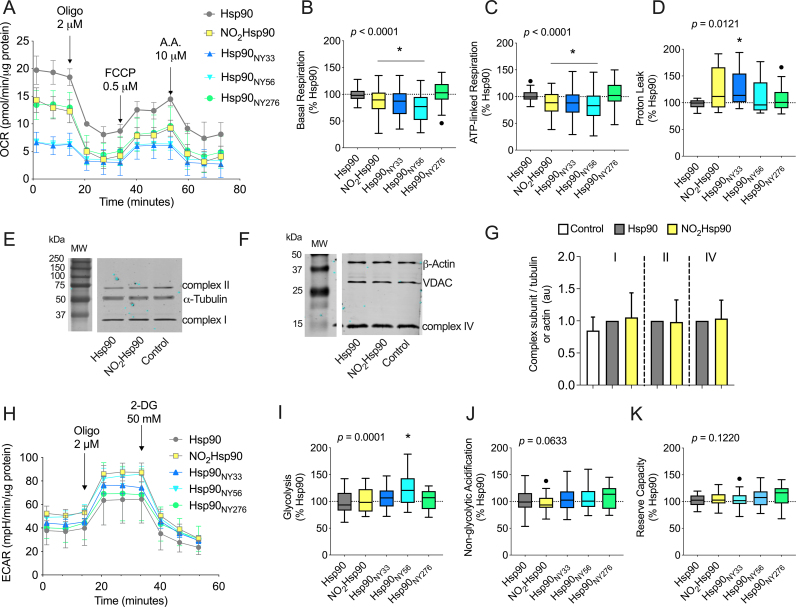
**Delivery of Site-specifically nitrated Hsp90 in normal Schwann cells recapitulates the metabolic reprogramming of schwannoma cells.** (A) Representative experiment showing the OCR of Schwann cells 24 h post-protein delivery in basal conditions, followed by the sequential addition of oligomycin (2 μM, oligo), FCCP (0.5 μM), and antimycin A (AA, 10 μM). (B–D) Analysis of the different OCR parameters post addition of the inhibitors. (E–G) Representative infrared Western blots of the steady state levels of (E) complex I (subunit NDUFA9) and II (subunit SDHA), and (F) complex IV (COX4 subunit) 24 h after delivery of the recombinant proteins. (G) Quantitation of the corresponding bands normalized against α-tubulin or β-actin signals. The columns represent the mean ± SD *versus* delivery of recombinant Hsp90 (n = 5–7). (H) Representative experiment showing the ECAR of Schwann cells 24 h post-protein delivery, measured by extracellular flux analysis in basal conditions and following the sequential addition of oligomycin (2 μM, oligo) and 2-deoxyglucose (50 mM, 2-DG). (I–K) Analysis of the different ECAR parameters following the sequential addition of the inhibitors, expressed as the percentage of the intracellular delivery of Hsp90. Data is shown using Tukey's representation with the median indicated in the box plot and outliers indicated with dots (n = 5 with 8 replicates). *p < 0.005 *versus* Hsp90 by one-way ANOVA with Dunnett's multiple comparisons post hoc test.

Next, we assessed whether supplementing Schwann cells with the different forms of nitrated Hsp90 could recapitulate the metabolic reprogramming observed in schwannoma cells. To this end, recombinant NO_2_Hsp90, Hsp90_NY33_, Hsp90_NY56_, or Hsp90_NY276_ were delivered into Schwann cells followed by assessment of mitochondrial activity and glycolysis by extracellular flux analysis. Intracellular delivery of NO_2_Hsp90 decreased basal and ATP-linked respiration compared to intracellular delivery of Hsp90 (Fig. 4A–C). However, there were no statistically significant changes in proton leak (Fig. 4D). We previously showed that peroxynitrite and tyrosine nitration decreased the levels of the mitochondrial oxidative phosphorylation complexes, which could explain the decrease observed in mitochondrial respiration. However, the intracellular delivery of NO_2_Hsp90 had no effect on the steady-state levels of complexes I, II and IV subunits in Schwann cells (Fig. 4E–G), suggesting that nitrated Hsp90 acts together with other yet unidentified nitrated protein(s) to regulate schwannoma cell metabolism.

To identify the residues responsible for these effects, Hsp90_NY33_, Hsp90_NY56_, or Hsp90_NY276_ were delivered into Schwann cells and mitochondrial activity and glycolysis measured. In contrast with the effect observed in schwannoma cells, nitration at either Y33 or Y56 decreased basal and ATP-linked mitochondrial respiration (Fig. 4A–C). Proton leak increased only with Hsp90_NY33_ compared to the delivery of Hsp90, as observed in schwannoma cells (Fig. 4D). On the other hand, intracellular delivery of Hsp90_NY276_ had no effect on mitochondrial activity (Fig. 4A–D). In parallel to the glycolytic effect observed in schwannoma cells, Hsp90_NY56_ increased glycolysis compared to the delivery of Hsp90 (Fig. 4H and I). Furthermore, none of the delivered protein impacted non-glycolytic acidification (Fig. 4H–J) or glycolytic reserve capacity (Fig. 4H–K). In summary, delivery of Hsp90 nitrated at Y33 decreased mitochondrial activity while Hsp90 nitrated at Y56 increased glycolysis, recapitulating the metabolic effects observed in schwannoma cells. Collectively, these results suggest that different forms of nitrated Hsp90 play distinct proliferative and metabolic roles in schwannoma cells.

### 2.5 P2X7R activation mediates the proliferative and glycolytic effects of Hsp90_NY56_ in schwannoma cells

**Fig. 5:**
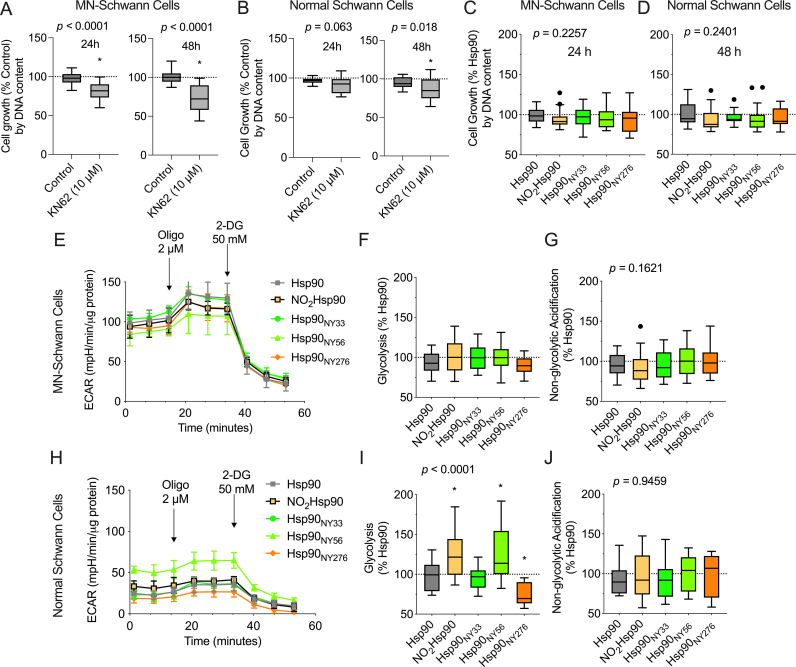
**Nitrated Hsp90 proliferative activity is mediated by activation of P2X7R.** (A) Schwannoma (MN-Schwann cells), and (B) normal Schwann cell growth was assessed in the presence and absence of the P2X7R inhibitor KN-62 (10 μM). (C–D) Recombinant proteins were intracellularly delivered to schwannoma and normal Schwann cells and the cells incubated for 24 and 48 h in the presence and absence of the P2X7R inhibitor KN62 (10 μM). The ECAR and glycolytic parameters of (E–G) schwannoma cells, and (H–J) normal Schwann cells were measured 24 h after delivery. Data is shown using Tukey's representation with the median indicated in the box plot and outliers indicated with dots (n = 3–8 with 8 replicates). *p < 0.05 *versus* Hsp90 by one-way ANOVA with Dunnett's multiple comparisons post hoc test.

We previously established that Hsp90_NY56_ activates the purinergic receptor P2X7 (P2X7R) in motor neurons and PC12 cells [19,20]. Other groups showed that activation of P2X7R increases glycolysis and tumor growth, suggesting that this form of nitrated Hsp90 may increase glycolysis and cell proliferation in schwannoma cells through activation of P2X7R [44]. We first assessed the role of P2X7R on schwannoma and normal Schwann cell growth. Schwannoma cells were sensitive to P2X7R inhibition, showing a significant decrease of ∼20 % and ∼30 % in cell growth when treated with the selective P2X7R inhibitor KN62 (10 μM) for 24 and 48 h, respectively (Fig. 5A). In contrast, a ∼10 % decrease in cell growth was observed in normal Schwann cells after 48 h incubation in the presence of the inhibitor (Fig. 5B), suggesting that P2X7R plays an important role supporting schwannoma cell proliferation and/or survival. We next assessed the role of P2X7R activation by nitrated Hsp90 in schwannoma and normal Schwann cell proliferation. Incubation with KN62 (10 μM) for 24 h or 48 h post-intracellular delivery of NO_2_Hsp90 or site-specifically nitrated Hsp90 in schwannoma and normal Schwann cells, respectively, abrogated the proliferative effect in both schwannoma cells (Fig. 5C) and normal Schwann cells (Fig. 5D).

To determine if the effect of Hsp90_NY56_ on glycolysis was P2X7R-dependent, we incubated both schwannoma and normal Schwann cells in the presence or absence of KN62 (10 μM) following the intracellular delivery of the site-specifically nitrated proteins. Incubation with KN62 abolished the effects of NO_2_Hsp90 and Hsp90_NY56_ on glycolysis in schwannoma cells (Fig. 5E–G) but not in normal Schwann cells (Fig. 5H–J). Surprisingly, the combination of intracellular Hsp90_NY276_ delivery and KN62 treatment decreased glycolysis, but only in normal Schwann cells (Fig. 5H–J). Collectively, these results suggest that the effects of site-specific nitrated Hsp90 on glycolysis may vary depending on the levels of nitrated Hsp90, the cell type, and context. However, in schwannoma cells, the glycolytic effects of site-specifically nitrated Hsp90 are P2X7R-dependent.

These results suggest that the effect of Hsp90_NY56_ on cell metabolism may at least partially depend on the cellular context, while its effect on proliferation is P2X7R-dependent.

### 2.6 The spatial distribution of nitrated Hsp90 in 3D-cell culture models is residue dependent

**Fig. 6:**
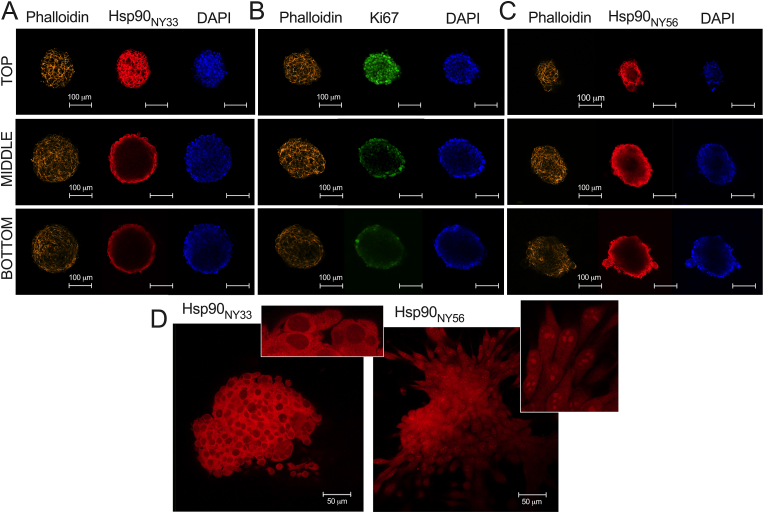
**Distinct forms of nitrated Hsp90 are differentially distributed in a three-dimensional cell culture model of schwannoma cells.** Representative confocal images of top, middle, and bottom optical sections of schwannoma cell clusters (∼200 μm diameter). The clusters were stained for F-actin (phalloidin, orange), the nuclear stain DAPI (blue), and for (A) Hsp90 nitrated at Tyr 33 (Hsp90_NY33_, red), (B) the nuclear proliferation marker Ki67 (green), and (C) Hsp90 nitrated at Tyr 56 (Hsp90_NY56_, red). (D) Cell clusters stained for Hsp90_NY33_ or Hsp90_NY56_ (red). Insets show higher magnification of a section of the corresponding image highlighting the subcellular distribution of the endogenously nitrated forms of Hsp90.

Given the distinct functions of Hsp90 when nitrated at Y33 *versus* Y56, we sought to determine the spatial distribution of these different forms of nitrated Hsp90 in three-dimensional (3D) cell culture models of schwannoma cells, using antibodies that specifically recognize either Hsp90_NY33_ or Hsp90_NY56_ [[19], [20], [21]]. Merlin-null schwannoma cells spontaneously form cell clusters when cultured at high density (Sup. Fig. 3A, real-time movie, and Sup. Fig. 3B and C). In cell clusters, Hsp90_NY33_ localized in the periphery, in the outer cellular layer (Fig. 6A), correlating with the proliferation marker Ki67 (Fig. 6B), whereas Hsp90_NY56_ was found homogenously distributed throughout the cell clusters, with a distribution correlating with that of the nuclear stain DAPI (Fig. 6C). Further, the intracellular distribution of Hsp90_NY33_ was cytosolic and mitochondrial (Fig. 6D, inset and [27]), whereas Hsp90_NY56_ was also found in nuclei (Fig. 6D, inset). These results suggest that an interplay in the levels of nitrated Hsp90 subpopulations with distinct activities could confer different advantages to specific cellular populations within a solid tumor.

## 3 Discussion

One of the functions of the cytoskeleton-associated tumor suppressor protein merlin, encoded by the *NF2* gene, is to sense cell to cell contact and initiate contact-dependent growth inhibition [[45], [46], [47]]. Loss of merlin function causes NF2-SWN, characterized by formation of schwannomas and other solid tumors (meningiomas and ependymomas) in the nervous system [10,48]. It was described that merlin-deficiency alters redox homeostasis [2,46]. We showed that compared with normal Schwann cells, schwannoma cells undergo a metabolic reprogramming characterized by increased glycolysis and decreased mitochondrial metabolism, and that this metabolic reprogramming is triggered by tyrosine nitration [27]. However, the identity of the nitrated protein(s) responsible for the pro-survival and/or proliferative and metabolic activities remained unknown.

Among the proteins that are a target for nitration, we discovered that nitration of Hsp90 induces a pathological gain-of-function in different cell types, associated with altered mitochondrial metabolism and induction of cell death [[19], [20], [21],^27^]. Using complementary approaches and methodologies, and carefully considering the stoichiometry of endogenous tyrosine nitration, we have identified nitrated Hsp90 as the first nitrated protein with proliferative activity in tumors. We discovered that doubling the intracellular levels of endogenous nitrated Hsp90 increased the proliferation of schwannoma cells at 12 and 24 h after delivery compared with Hsp90, following a distribution that adjusted well to an exponential plateau growth, in contrast with the classic exponential growth observed after delivering Hsp90. Schwannoma cells show upregulation of stem cell markers such as SOX2 [49], CD133, and OCT4 [50]. In mouse embryonic stem cells, activation of P2X7R accelerates the entry into the cell cycle by promoting entry into S phase [51]. An intriguing possibility to explore in the future is that the delivered nitrated Hsp90 may transiently increase proliferation of a subpopulation of cells undergoing a specific stage of the cell cycle, or cells with a stem-like phenotype, sensitive to nitrated Hsp90 stimulus. When the effect of the stimulus declines, either by nitrated Hsp90 degradation or dilution due to cell division, some of these cells may undergo asymmetric division and differentiation.

Genetic code expansion enables the production of site-specific nitrated Hsp90 in bacteria but not yet in mammalian cells, thus the effects triggered by the intracellular delivery of nitrated Hsp90 in mammalian cells are transient. However, we were able to detect the delivered recombinant protein 48 h after intracellular delivery in normal Schwann cells (Sup. Fig. 1E). Supplementing normal Schwann cells with levels of nitrated Hsp90 comparable to those endogenously found in schwannoma cells and NF2-SWN schwannomas induced a ∼20 % increase in cell proliferation at 48 h, similar to the ∼15–20 % increase observed in schwannoma cell proliferation 24 h after delivery. The lag in nitrated Hsp90 proliferative effect observed in normal Schwann cells *versus* schwannoma cells could be explained by the lower levels of nitrated protein with proliferative activity in normal Schwann cells, as these cells do not contain endogenously nitrated Hsp90 [27]. Additionally, the doubling time of normal Schwann cells is longer than that of schwannoma cells (∼60–72 h *versus* ∼28–30 h, respectively), which would contribute to the delayed effect, while also delaying dilution of the delivered protein. Further, it is possible that the turnover of Hsp90 is altered in schwannoma cells compared to normal Schwann cells. However, the data suggests that nitrated Hsp90 increases cell proliferation. Therefore, nitrated Hsp90 is a potential tumor-directed therapeutic target in schwannoma cells, as targeting nitrated Hsp90 impacts tumor cells without affecting normal, healthy cells which do not contain detectable levels of nitrated proteins.

We previously showed that Hsp90, when nitrated at Y33, forms a mitochondrial complex that decreases mitochondrial activity [21], and that nitration of this residue induces cell death in motor neurons [19] but not in PC12 cells [20,21]. On the other hand, using multiple complementary approaches we showed that nitration of Hsp90 at Y56 induces motor neuron and PC12 cell death through the activation of P2X7R but triggers different mechanisms downstream of the receptor [19,20]. Together, these observations suggest that there is a complex interplay between the two functionally distinct forms of nitrated Hsp90, Hsp90_NY33_ and Hsp90_NY56_, depending on the conditions and the cell type. P2X7R is a homotrimer expressed in multiple cell types that forms a non-specific cationic channel [52]. The receptor is part of a larger protein complex of varying composition that regulates its activation. P2X7R has 3 ATP binding sites, occupancy of 1 or 2 of favors channeling opening for permeability to Na^+^, Ca^2+^ and K^+^, followed by a transition to the desensitized state. During prolonged activation, when all 3 binding sites are occupied, the receptor forms a pore and becomes resistant to inactivation. These differences in P2X7R activation state allow the receptor to play a variety of cellular roles ranging from induction of cell proliferation to cell death, depending on the context and cell-type [44]. In tumor cells, it has been shown that activation of P2X7R increases aerobic glycolysis and promotes tumor cell proliferation in different cancer types, including prostate cancer and neuroblastoma [44,53], and tumor growth *in vivo* [54]. We showed that Hsp90 nitrated at Y56 associates with the P2X7R complex and activates the receptor [19]. Thus, activation of P2X7R by nitration of Hsp90 at Y56 in schwannomas *in vivo* could increase aerobic glycolysis and cell proliferation, while nitration at Y33 could decrease mitochondrial metabolism, posing nitrated Hsp90 as a metabolic switch in tumor cells. Indeed, we found that both Hsp90_NY33_ and Hsp90_NY56_ supported cell proliferation in schwannoma and normal Schwann cells and regulated their metabolic phenotype. Their proliferative effects were abrogated by P2X7R inhibition [55]. P2X7R is also found in the mitochondrial outer membrane [56], which may explain how the Hsp90_NY33_ effects are mediated by P2X7R activation. In summary, both forms of nitrated Hsp90 directly or indirectly activate P2X7R to support cell proliferation.

Metabolically, Hsp90_NY33_ decreased basal and ATP-linked mitochondrial respiration, while increasing proton leak in both schwannoma and normal Schwann cells. Surprisingly, Hsp90_NY56_ induced a decrease in basal and ATP-linked respiration but only in Schwann cells and without affecting proton leak, suggesting that these two forms of nitrated Hsp90 regulate mitochondrial activity through different mechanisms yet to be established. We showed that Hsp90 nitrated at Y33 associates with the outer mitochondrial membrane, forming a complex that decreases the activity of mitochondrial complex I through IV [21]. It is possible that Hsp90_NY33_ associates with mitochondrial proteins such as the voltage-dependent anion channel (VDAC) on the outer mitochondrial membrane. VDAC together with the adenine nucleotide translocase (ANT) in the inner mitochondrial membrane control transport of ADP and ATP, thus regulating the ADP/ATP ratio [57]. By acting on VDAC/ANT activity, Hsp90_NY33_ could indirectly regulate the activity of the oxidative phosphorylation. We also showed that peroxynitrite and tyrosine nitration decrease the levels of oxidative phosphorylation complexes [27]. However, we did not observe changes in the levels of the mitochondrial complexes after intracellular delivery of nitrated Hsp90, an indication that nitrated Hsp90 is not the sole nitrated protein regulating mitochondrial metabolism in schwannoma cells.

Interestingly, P2X7R inhibition abrogated the glycolytic effect of Hsp90_NY56_ only in schwannoma cells, indicating that the effects of Hsp90_NY56_ may depend on the composition of the P2X7R complex, and/or that nitrated Hsp90 signaling may crosstalk with pathways that are dysregulated in the schwannoma cell culture model. P2X7R activation stimulates the proliferation of a variety of cell types, including glial cells [[58], [59], [60]] and it is well-established that the composition and regulation of the P2X7R complex is both cell-type and condition specific [[61], [62], [63], [64]]. We and others showed that Hsp90 within the complex represses P2X7R activity [65,66], thus it is possible that Hsp90 nitration may alter the specific composition of the P2X7R complex in schwannoma cells as to render it more susceptible to activation. Collectively, these results suggest that targeting P2X7R could present a promising new therapeutic avenue for NF2-SWN patients.

Unexpectedly, Hsp90_NY276_ increased proliferation but only in normal Schwann cells, and had no apparent effect on energy metabolism, suggesting that the proliferative effect of this form of nitrated Hsp90 is different from that of Hsp90_NY33_ or Hsp90_NY56_. It is possible that schwannoma cells contain high levels of Hsp90 endogenously nitrated at Y276, which could mask the proliferative effect of the delivered recombinant nitrated protein in these cells. Moreover, delivery of Hsp90_NY276_ into normal Schwann cells decreased glycolysis but only in the presence of KN62, which may explain why inhibition of P2X7R after delivery of Hsp90_NY276_ abolished its proliferative effect in these cells. Using complementary approaches, we showed that the P2X7-dependent pathogenic functions of nitrated Hsp90 in the induction of apoptosis in motor neurons and PC12 cells are triggered by a calcium influx due to P2X7R activation [19,20]. In agreement with our previous findings, the potent non-competitive antagonist of P2X7R, KN62 (IC 50 = 15 nM), abolished the effect proliferative effects of all forms of nitrated Hsp90,suggesting that their proliferative effect may be calcium dependent. KN62 is also a weak inhibitor of CAMK II (IC50 = 900 nM). Interestingly, CAMK II is a major effector of calcium signaling, activated downstream of P2X7R. In hippocampal neurons, P2X7R signaling through CAMKII regulates axon growth [67], and in neuroblastoma cells, activation of CaMK II by P2X7R inhibits neurite growth [68], and induces cell proliferation in the absence of serum [69], suggesting that CAMKII may be a component of the P2X7R signaling pathway in schwannoma cells. Since different forms of nitrated Hsp90 play distinct metabolic activities, changes in the levels of specific forms could confer subpopulations of cells within a tumor different metabolic advantage. For example, in schwannoma cell clusters we found that while high levels of Hsp90_NY33_ were mainly confined to the outer cellular layer, Hsp90_NY56_ was homogenously distributed throughout the cell cluster. Intracellularly, the spatial distribution of Hsp90_NY33_ was cytosolic and mitochondrial, while Hsp90_NY56_ also colocalized with nuclei. Hsp90_NY56_ association with nuclei could indicate regulation of transcription factor(s). For example, RE1-silencing transcription factor (REST) is a known Hsp90 client, and REST itself is a repressor of genes that promote cell death [70,71]. Considering the differential localization, abundance, and metabolic activity of the different forms of nitrated Hsp90, an interesting hypothesis to explore in the future, proposing a dual role for the nitrated chaperone in tumor growth, is that in the highly proliferative cell layer with access to oxygen and nutrients, Hsp90_NY33_ decreases oxygen consumption while Hsp90_NY56_ increases glycolysis to produce metabolic intermediates that support active cell proliferation. Nitration of Hsp90 at Y33 in the outer layer also allows oxygen to penetrate further within the cellular core, while nitration at Y56 increases glycolysis to support cell survival at lower oxygen tensions.

The differential location and activities of nitrated Hsp90 could be explained by a selective change in the Hsp90 interactome due to differential changes in structure and/or post-translational modifications, depending on the tyrosine that is nitrated. It is well established that post-translational modifications regulate Hsp90 interactome. For example, Y33 and Y56 are also target for phosphorylation [72,73]. While no function associated with phosphorylation of Y56 has been described to-date, cell cycle-associated phosphorylation on Y24 by Swe1 in budding yeast (Y33 in human Hsp90β) allows binding of Hsp90 to a select set of co-chaperones and client proteins [73]. Tyrosine nitration and phosphorylation seem to be mutually exclusive; if a tyrosine is nitrated it cannot be phosphorylated and nitration may not mimic the effects of phosphorylation. Further, nitration of a tyrosine residue may facilitate phosphorylation of other residues in a protein and vice versa, highlighting a complex interplay between these two post-translational modifications in cell signaling [[74], [75], [76], [77]].

In conclusion, we established nitrated Hsp90 as a major player in schwannoma metabolic reprogramming and cell proliferation. This is the first demonstration of residue-specific nitration of a protein leading to forms of the nitrated protein with distinctly different localization and pathologically relevant activity. Hsp90 is endogenously nitrated in schwannomas but not in normal Schwann cells. In addition, we showed that preventing tyrosine nitration decreases viability in schwannoma cells but has no effect in normal Schwann cells, suggesting that nitrated proteins are relevant to tumor cell survival/proliferation [27]. Thus, the selective targeting of nitrated Hsp90 could be an effective tumor-directed therapeutic approach, alone or in combination strategies. Together, these observations support nitrated Hsp90 and P2X7R as novel therapeutic targets for treatment of NF2-SWN and other solid tumors where Hsp90 is nitrated. Further, nitrated Hsp90 is one of several nitrated proteins found in tumors, suggesting that additional nitrated protein(s) may also function as disease drivers, opening an exceptional opportunity for the development of a new category of tumor-directed targets for therapeutic development.

## 4 Materials and methods

### 4.1 Human and mouse schwann cell cultures

Low passage normal human Schwann cells (Cat. No. 1700, ScienCell Research Laboratories, Carlsbad, CA, USA) were cultured on CellBIND dishes (Corning, Fisher Scientific, Hampton, NH, USA) in ScienCell Schwann cell culture media, as described previously [27]. Mouse merlin-null schwannoma cells were generated in the laboratory of Dr. Cristina Fernandez-Valle by deleting the sequence coding for exon 2 in the *Nf2* gene in Schwann cells purified from sciatic nerve of *Nf2*^*flox2/flox2*^ mice, as described previously [78]. These cells form tumor allografts in mice [79]. All cultures were routinely tested for mycoplasma contamination (LookOut Mycoplasma PCR detection kit, Sigma-Aldrich, St. Louis, MO, USA). The absence of merlin in schwannoma cells was confirmed regularly by Western blot (1:2000, Cat. No. 12888, Cell Signaling Technology, Danvers, MA, USA).

### 4.2 Expression and purification of recombinant human Hsp90β and genetic code expansion (GCE) mutants

As described previously, the human Hsp90β sequence was optimized and cloned into a pBad/Myc-Hisx6 vector (Invitrogen, Thermo Fisher Scientific, Waltham, MA, USA) [19]. Recombinant protein carrying a Myc-Hisx6 tag was expressed after induction of the bacterial culture with 0.2 % l-arabinose for 2 h at 37 °C and 180 rpm. Recombinant wild-type Hsp90β was purified from the bacterial culture using a Ni-NTA purification system (Invitrogen) following the manufacturer's instructions. The insertion of nitrotyrosine residues at specific positions was performed using genetic code expansion (GCE), as described previously [19,80]. Briefly, the codons coding for each of the five tyrosine residues prone to nitration in human Hsp90β were independently replaced by an amber UAG stop codon. Nitrotyrosine was incorporated into the recombinant proteins by an orthogonal nitrotyrosine-bearing suppressor tRNA that recognizes the UAG stop codon and respective engineered aminoacyl-tRNA synthetase. Proteins modified by GCE were expressed and purified using the same induction and purification methods used for recombinant Hsp90β.

### 4.3 Intracellular protein delivery

The intracellular protein delivery was performed as previously described [[19], [20], [21]]. Briefly, human recombinant Hsp90β, peroxynitrite-treated human recombinant Hsp90β, or the GCE Hsp90β mutants, a 200 μL mixture containing 10 μg of protein in 100 μL DPBS and 4 μL of the lipophilic cell permeation agent Chariot (Active Motif, Carlsbad, CA, USA) in 100 μL ddH_2_O was incubated at room temperature for a total of 30 min. A pellet containing 1 × 10^6^ schwannoma or normal Schwann cells was resuspended in the protein/Chariot mixture, immediately followed by the addition of 400 μL serum-free DMEM. Following 1 h incubation at 37 °C in 5 % CO_2_ atmosphere and humidity with resuspension at 20 min intervals, 1.0 mL Schwann cell culture media containing 16 % fetal bovine serum was added, followed by another 1 h incubation with resuspension at 20 min intervals. Following this second incubation, the cells were plated for further experiments, such as 7.5 × 10^3^ cells per well of 96 well CellBIND plates to measure cell growth or 2.0 × 10^4^ per well of poly-l-lysine coated 96-well plates to perform extracellular flux analysis.

### 4.4 Oxygen consumption rate

Oxygen consumption rate (OCR) was measured in schwannoma and normal Schwann cells 24 h post protein delivery using an XF96 Extracellular Flux Analyzer (Agilent Technologies, Santa Clara, CA, USA), as previously described [27]. Immediately after intracellular delivery, cells were seeded into poly-l-lysine coated 96-well plates at a density of 2.0 × 10^4^ cells per well and incubated for 24 h at 37 °C in 5 % CO_2_ and humidity. At the start of the experiment, cell culture media was replaced with seahorse basal medium (pH 7.4, Cat. no. 103334-100, Agilent Technologies) supplemented with 25 mM glucose (Cat. no. 103577-100, Agilent Technologies) and 1 mM l-glutamine (Cat. no. 103579-100, Agilent Technologies) and cells were incubated for 1 h at 37 °C with humidity. The basal OCR was measured followed by the sequential addition of oligomycin (1 μM, Cat. no. 75351-5 MG, Sigma-Aldrich, St. Louis, MO, USA), carbonyl cyanide *p*-trifluoromethoxy phenylhydrazone (FCCP, 0.5 μM, Cat. no. C2920, Millipore Sigma, Burlington, MA, USA), and antimycin A (10 μM, Cat. no. A8674, Millipore Sigma). The OCR from each well was normalized to the protein content of that well, quantified by Lowry assay, and the non-mitochondrial respiration following the addition of antimycin A was subtracted from all OCR measurements. The P2X7R inhibitor KN62 (10 μM, Cat. no. S7422, Selleck Chemicals) was dissolved in DMSO and administred at the final concentration indicated. Cell culture DMSO concentration did not exceed 0.01 %.

### 4.5 Glycolysis stress test

We measured the extracellular acidification rate in mouse schwannoma and normal human Schwann cells 24 h post protein delivery using the XF96 Extracellular Flux Analyzer (Agilent Technologies), as previously described [27]. The cells were seeded at a density of 2.0 × 10^4^ cells per well of a poly-l-lysine coated 96-well plate. To measure the extracellular acidification rate, basal seahorse medium (Cat. no. 103334-100, Agilent Technologies) was supplemented with 1 mM l-glutamine (Cat. no. 103579-100, Agilent Technologies) and 25 mM glucose (Cat. no. 103577-100, Agilent Technologies). Extracellular acidification rate was measured following the sequential injection of 1 μM oligomycin (Cat. no. 75351-5 MG, Sigma-Aldrich, St. Louis, MO, USA), and 50 mM 2-deoxyglucose (Cat. no. D8375-5G, Sigma-Aldrich, St. Louis, MO, USA) to inhibit glycolysis.

### 4.6 Quantitative dot blot, slot blot, and western blot analysis

The immunoblot analysis was performed as described previously with minor modifications [19]. Briefly, twenty μg of total schwannoma cell homogenate, or the indicated mass, was loaded directly onto a precut 0.2 μm nitrocellulose membrane (Cat. no. 1620168, Bio-Rad Laboratories, Germany) captured within the dot blot or slot blot apparatus (Model no. DHM-96, Scie-Plas, United Kingdom). Standard curves of site-specifically nitrated Hsp90, either Hsp90_NY33_, or Hsp90_NY56_ (0–200 ng) were included. The negative control consisted of 20 μg total normal Schwann cell homogenate. The nitrocellulose membrane was rinsed three times prior to protein incubation. The membranes were then blocked using TBS Odyssey Blocking Buffer (Cat. no. 927–60001, Li-Cor Biosciences, Lincoln, NE, USA), and incubated overnight at 4 °C with their indicated in-house developed primary antibodies against nitrated Hsp90, or an anti-myc tag antibody [[19], [20], [21]]. IRDdye secondary goat antibodies (Li-Cor Biosciences), anti-mouse (680RD, Cat. no. 925–68070), and anti-rabbit (800CW, Cat. no. 925–32211) were used at a 1:20,000 dilution. All dot blots were visualized and quantified with the Odyssey System (Li-Cor Biosciences). After densitometric analysis of dots, protein content was calculated as percent of total Hsp90 cell content. Western blotting was performed similarly to the dot and slot blots, except 75 μg of total cell homogenate was loaded.

### 4.7 Cell growth assays

For the colorimetric cell growth assays, cells were seeded at a density of 10,000 cells per well in a 96-well CellBind plate (Cat. no. 3300, Corning) and incubated for either 24 h or 48 h for schwannoma and normal Schwann cells, respectively. At their final time point, cells were rinsed once with PBS and incubated with 50 μl 0.2 % crystal violet (Cat. no. F906–03, J.T.Baker, NJ, USA) in 20 % methanol per well for at least 20 min. Wells were rinsed with ice cold distilled water before solubilizing the fixed cells with 200 μL of 1 % SDS. Absorbance at 595 nm was measured for each well (SpectraMAX Plus, Molecular Devices, CA, USA). The MTS assay was performed using the CellTiter 96® AQueous One Solution Cell Proliferation Assay (Cat. no. G3580, Promega), according to the manufacturer's instructions. Cell growth is expressed as percentage of control. To perform the cell growth curves, 5000 cells per well were plated in a black 96-well plate with clear bottom (Cat. no 655096, Greiner Bio-One) coated with poly-l-lysine. The cells were fixed at 4, 12 and 24 h post-delivery, and incubated with the nuclear stain 4′,6-diamidino-2-phenylindole (DAPI; Cat. no. D1306, ThermoFisher Scientific, 300 nM). All nuclei per well were imaged and counted using a Cytation 5 instrument equipped with Gen5 software (Biotek, Agilent). The seeding density was adapted to ensure the cells would not become overconfluent at the 24 h or 48 h timepoints for the colorimetric assays and optimized for cell counting. Cell proliferation is expressed as percentage of 4 h post-delivery (immediately after cell attachment).

### 4.8 Immunohistochemistry

Schwannoma cells were seeded into 8-chamber slides (Cat. no. 177445, Lab-Tek, Grand Rapids, MI, USA) at a density of 12.5 x 10^4^ cells per well and incubated until cell clusters spontaneously formed. After removal of cell culture medium, cells were fixed with 4 % paraformaldehyde/0.2 % glutaraldehyde for 30 min at room temperature. Cells were incubated with DAPI and phalloidin-Alexa Fluor 647 (Cat. no. A22287, ThermoFisher Scientific) as counterstains to visualize the cells. Cells were stained with antibodies specific for Ki-67 (1:800 dilution, Cat. no. 9449, Cell Signaling), or in-house developed antibodies specific for Hsp90 nitrated at Tyr 33 or Tyr 56 (dilution 1:200) [[19], [20], [21]]. Slides were rinsed with DPBS three times for 5 min and ProLong Gold Antifade mounting solution added (Cat. no. P36934, ThermoFisher Scientific). Fluorescence images were collected using an inverted confocal microscope (Cat. no. LSM780 NLO, Carl Zeiss AG, Dublin, CA, USA).

### 4.9 Statistical analysis

Statistical analysis was performed using GraphPad Prism (GraphPad Software Inc., San Diego, CA, USA)*.* When populations followed a normal distribution, the two-tailed Student's t-test determined statistical significance of two groups, while a one-way ANOVA with Dunnett's multiple comparisons post hoc test determined statistical significance in the case of more than two groups. For those populations not possessing normal distributions, either a Wilcoxon (two groups) or Kruskal-Wallis (more than two groups) with Dunn's multiple comparisons nonparametric tests were used. The data was visualized using Tukey's boxplots, with the median indicated and outliers illustrated with dots. Statistical significance was set at *p* ≤ 0.05.

## CRediT authorship contribution statement

**Isabelle E. Logan:** Writing – original draft, Visualization, Validation, Supervision, Methodology, Investigation, Funding acquisition, Formal analysis, Data curation, Conceptualization. **Kyle T. Nguyen:** Writing – review & editing, Investigation, Data curation. **Tilottama Chatterjee:** Writing – review & editing, Investigation. **Bhagyashree Manivannan:** Investigation. **Ngozi P. Paul:** Investigation. **Sharon R. Kim:** Investigation. **Evelyn M. Sixta:** Investigation. **Lydia P. Bastian:** Investigation. **Carrie Marean-Reardon:** Writing – review & editing, Investigation. **Matthias A. Karajannis:** Writing – review & editing, Resources. **Cristina Fernández-Valle:** Writing – review & editing, Resources. **Alvaro G. Estevez:** Writing – review & editing, Resources, Methodology. **Maria Clara Franco:** Writing – review & editing, Visualization, Supervision, Resources, Project administration, Methodology, Investigation, Funding acquisition, Formal analysis, Data curation, Conceptualization.

## Declaration of competing interest

The authors declare that they have no known competing financial interests or personal relationships that could have appeared to influence the work reported in this paper.

## Data Availability

Data will be made available on request.
