# Supplementary material for: Selective nitration of Hsp90 acts as a metabolic switch promoting tumor cell proliferation
Source: Redox Biol. 2024 Jun 19;75:103249. doi: 10.1016/j.redox.2024.103249 (PMC11261529; doi:10.1016/j.redox.2024.103249)
Supplement: Multimedia component 1 [file mmc1.pdf]

Supporting Information for
Selective Nitration of Hsp90 Acts as a Metabolic Switch Promoting Tumor Cell Proliferation.

Isabelle E. Logan[1,2], Kyle T. Nguyen[1], Tilottama Chatterjee[1], Bhagyashree Manivannan[2], Ngozi P. Paul[2], Sharon R. Kim[1], Evelyn M. Sixta[1], Lydia P. Bastian[1], Carrie Marean-Reardon[1], Matthias A. Karajannis[3], Cristina Fernández-Valle[4], Alvaro G. Estevez[1,5], Maria Clara Franco[1,2,5*]

[1] Department of Biochemistry and Biophysics, College of Science, Oregon State University, Corvallis, OR 97331, USA.
[2] Center for Translational Science, Florida International University, Florida 34987, USA.
[3] Department of Pediatrics, Memorial Sloan Kettering Cancer Center, New York, NY 10065, USA
[4] Burnett School of Biomedical Sciences, College of Medicine, University of Central Florida, Orlando, FL 32827, USA.
[5] Department of Cellular and Molecular Medicine, Herbert Wertheim College of Medicine, Florida International University, Florida 33199, USA.

*Maria Clara Franco.
Email: marfranc@fiu.edu
Corresponding author address: 11350 SW Village Pkwy, Port St. Lucie, FL, 34987.

This file includes:

Figures S1 to S3

Other supporting materials for this manuscript include the following:

Movie S1

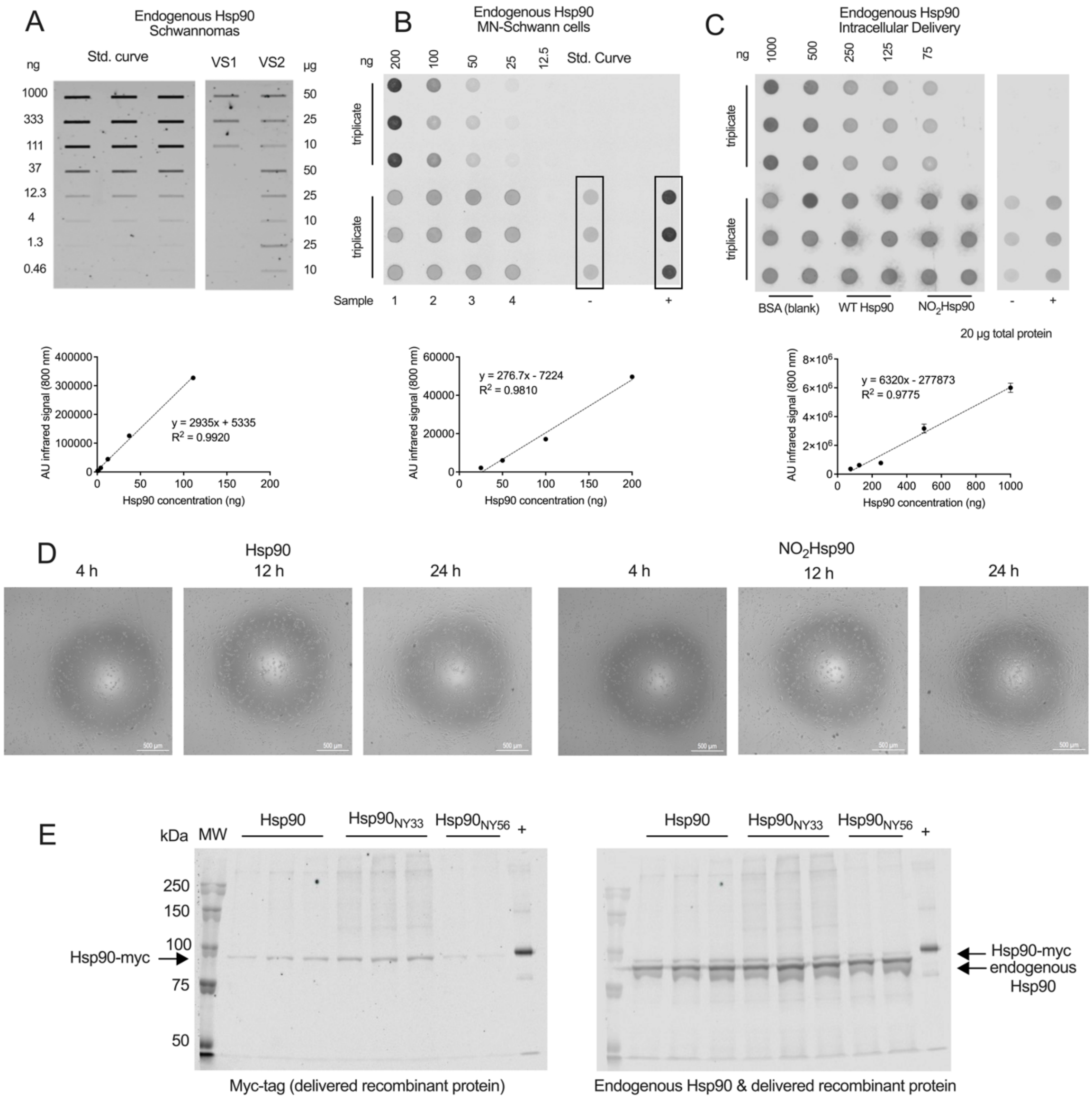

Figure S1. Quantitation of the endogenous levels of Hsp90 in NF2 vestibular schwannomas and schwannoma cells and detection of recombinant protein delivered into Schwann cells. Endogenous Hsp90 levels were assessed in: (A) Vestibular schwannomas (VS) from NF2 patients by infrared quantitative slot blot, (B) schwannoma cells (MN-Schwann cells) by infrared quantitative dot blot using an anti-Hsp90α/β antibody (800 nm channel), and (C) schwannoma cells following intracellular delivery of the recombinant forms of Hsp90. Recombinant NO_2Hsp90 was used to perform the standard (Std.) curve. (D) Representative phase contrast images (100X) of wells containing schwannoma cells at the indicated timepoints post-intracellularly delivery of Hsp90 or NO_2Hsp90. The scale bar represents 500 μm. (E) Representative infrared western blot showing the detection of recombinant protein using antibodies against the myc-tag (left) and total Hsp90 (right) 48 h after intracellular delivery in normal Schwann cells.

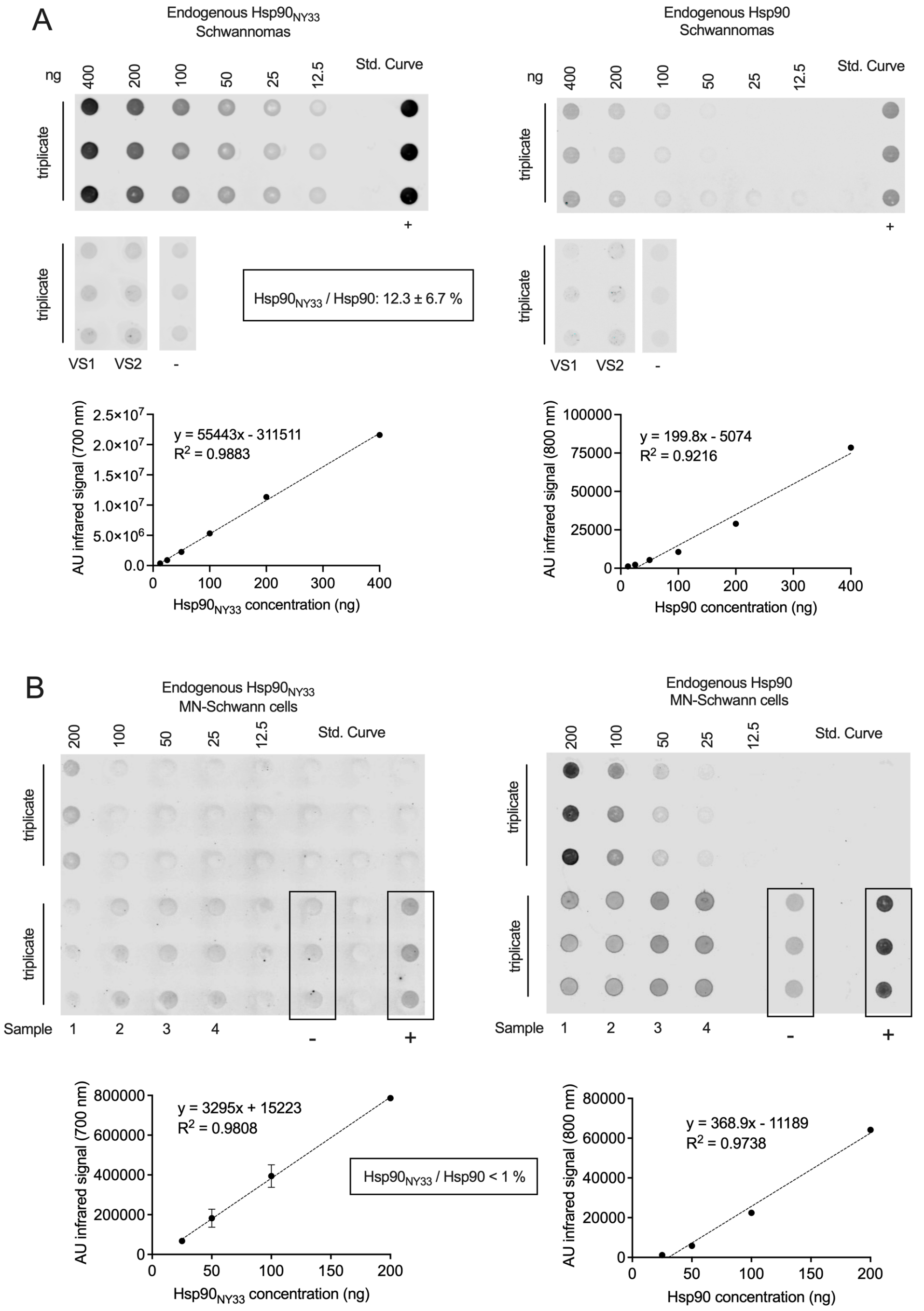

Figure S2. Level of Hsp90 nitrated on Y33 *vs.* total Hsp90 in VS from NF2-SWN tumor resections and schwannoma cells. (A) and (B) $Hsp90_{NY33}$ levels were assessed by infrared quantitative dot blot (700 nm channel) using a monoclonal antibody against Hsp90 nitrated at Y33 developed in-house. Total levels of endogenous Hsp90 were determined using an anti-Hsp90α/β antibody (800 nm channel). Recombinant $Hsp90_{NY33}$ was used to perform the standard (Std.) curve.

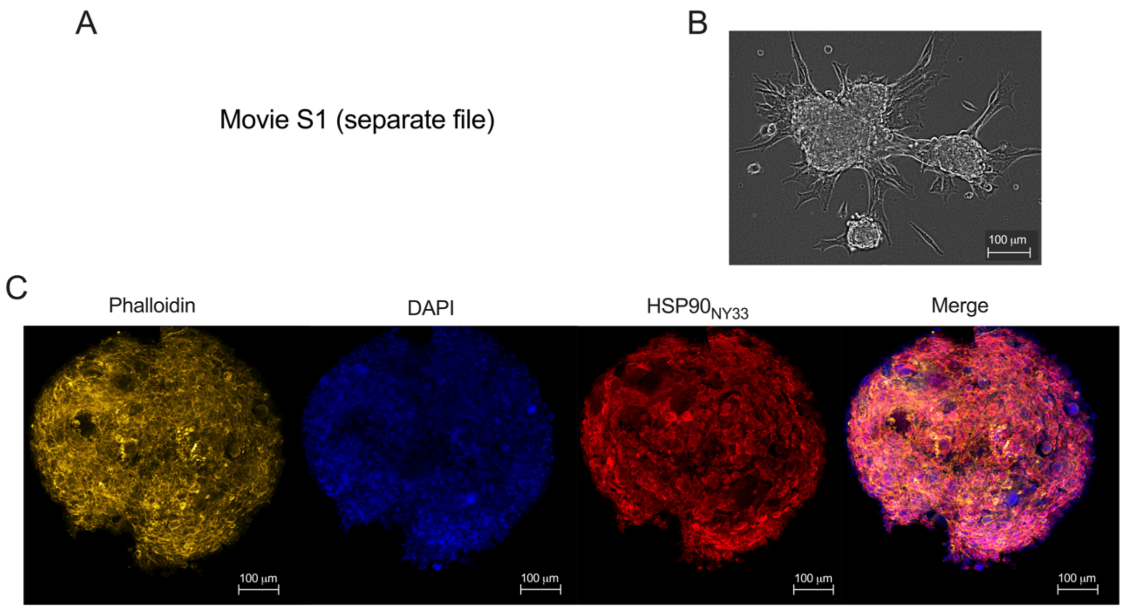

Figure S3. Schwannoma cells form clusters in culture. (A) Real-time movie of schwannoma cell cluster formation. (B) Cell cluster formed after 24h. (C) Top view of a cell cluster after 3 days in culture stained for phalloidin (purple), DAPI (blue) and $Hsp90_{NY33}$ (red). Merge indicates the overlapping of the three fluorescence signals.

Movie S1 (separate file). Real-time movie of schwannoma cell cluster formation.
